# Attitudes towards HPV testing: a qualitative study of beliefs among Indian, Pakistani, African-Caribbean and white British women in the UK

**DOI:** 10.1038/sj.bjc.6600686

**Published:** 2003-01-28

**Authors:** K McCaffery, S Forrest, J Waller, M Desai, A Szarewski, J Wardle

**Affiliations:** 1 Health Behaviour Unit, Department of Epidemiology and Public Health, Cancer Research UK, Royal Free and University College Medical School, London, UK; 2 Cytopathology Department, Manchester Cytology Centre, Christie Hospital and Holt Radium Institute, Kinnaird Road, Withington, Manchester M20 4BX, UK; 3 Department of Epidemiology, Mathematics and Statistics, Cancer Research UK, 44 Lincoln's Inn Fields, London WC2A 3PX, UK

**Keywords:** HPV testing, psychosocial impact, cervical screening

## Abstract

This study examined attitudes to human papillomavirus (HPV) testing among a purposively selected sample of women from four ethnic groups: white British, African Caribbean, Pakistani and Indian. The design was qualitative, using focus group discussion to elicit women's attitudes towards HPV testing in the context of cervical cancer prevention. The findings indicate that although some women welcomed the possible introduction of HPV testing, they were not fully aware of the sexually transmitted nature of cervical cancer and expressed anxiety, confusion and stigma about HPV as a sexually transmitted infection. The term ‘wart virus’, often used by medical professionals to describe high-risk HPV to women, appeared to exacerbate stigma and confusion. Testing positive for HPV raised concerns about women's sexual relationships in terms of trust, fidelity, blame and protection, particularly for women in long-term monogamous relationships. Participation in HPV testing also had the potential to communicate messages of distrust, infidelity and promiscuity to women's partners, family and community. Concern about the current lack of available information about HPV was clearly expressed and public education about HPV was seen as necessary for the whole community, not only women. The management of HPV within cervical screening raises important questions about informed participation. Our findings suggest that HPV testing has the potential to cause psychosocial harm to women and their partners and families.

Major changes are taking place in cervical screening. The development of sensitive and cost-effective tests for high-risk types of the human papillomavirus (HPV) (Cuzick and Sasieni, 1997; Kim *et al*, 2002; Mandelblatt *et al*, 2002), the primary causal agent of cervical cancer (Munoz *et al*, 1992; Schiffman, 1993; Bosch *et al*, 1995), suggests that HPV testing in triage and primary screening may be a feasible and attractive adjunct to conventional cytological screening (Cuzick *et al*, 2000; Solomon *et al*, 2001; Sasieni and Cuzick, 2002). The UK Department of Health, the Medical Research Council/National Health Service and the US National Cancer Institute are currently running trials of HPV screening for women at triage (UK National Screening Committee, 2000; Schiffman and Adrianza, 2000). Results of the US trial have recently been published and provide support for HPV testing among women with borderline smears (atypical cells of undetermined significance, ASCUS) (Solomon *et al*, 2001; Stoler, 2002; Sherman *et al*, 2002). In response, the Bethesda 2001 Consensus Guidelines for the Management of Women with Cervical Cytological Abnormality have recommended HPV testing for women with ASC-US (ASCUS) (Wright *et al*, 2002). Pressure to adopt HPV testing within cervical screening in the UK and other countries is steadily increasing.

Although HPV testing in cervical screening offers potential public health benefits, it also raises some difficult issues for women who undergo screening. As a sexually transmitted infection (STI), HPV is extremely common among the sexually active population, but awareness of the role of the virus may bring stigma to cervical cancer, and resurrect social and psychological barriers to screening that impact negatively on women. Screening for a sexually transmitted agent has important implications (Duncan *et al*, 2001), with particular significance for those in long-term monogamous relationships or within groups where lifelong monogamous relationships are more strongly advocated, such as some religious and ethnic minority groups (Elam *et al*, 1999). To date there have been few studies examining psychosocial implications of HPV testing and none with women of varying ethnicity.

This study examines attitudes to HPV testing within primary cervical screening among a sample of women purposively selected to provide contrasting religious and cultural beliefs and practices, which could influence attitudes and experiences related to HPV testing. Since the impact of HPV testing is under-researched, may be complex, and importantly is unpredictable, a qualitative methodology was selected to explore the diversity of issues surrounding HPV using an approach grounded in participants' perspectives.

## Participants and Method

### Participants

A total of 71 women (aged 20–59 years) eligible for cervical screening within Greater Manchester were recruited from social and community groups by ethnically matched community researchers, who conducted focus group discussions. Participants from four ethnic groups (self-identified as white British, African Caribbean, Indian and Pakistani), varying in age, marital/partner status and socioeconomic position (measured via education), were selected using principles of purposive sampling, a method of nonrandom sampling of participants possessing certain characteristics, to provide a range of demographic backgrounds and experiences of interest to the research area (see Table 1

**Table 1:** Sociodemographic characteristics

	**African-Caribbean (*n*)**	**Indian (*n*)**	**Pakistani (*n*)**	**White British (*n*)**	**Total *n* (%)**
Mean age	35	43	41	39	40
(range)	(20–58)	(20–59)	(31–56)	(26–55)	(20–59)
					
*Born in UK*					
Yes	10	2	3	15	30 (42.9)
No	6	17	17	1	41 (58.1)
					
*Country of education*					
UK	10	1	4	16	28 (43.1)
Outside UK	3	18	15	0	33 (50.8)
Both	3	0	1	0	4 (6.2)
					
*Age left full-time education*					
15 and under	5	1	2	3	11 (15.5)
16–18	6	7	8	12	33 (46.5)
19 and over	5	10	9	1	25 (35.2)
*Missing*	0	1	1	0	2 (2.8)
					
*Marital status*					
Married/living with partner	2	15	15	12	44 (62)
Single	12	2	3	3	20 (28.2)
Divorced/separated/widowed	2	2	2	1	7 (9.8)
					
*Ever attended a smear test*					
Yes	16	17	18	15	66 (93.0)
No	0	2	2	1	5 (7.0)
					
Total interviewed sample	16	19	20	16	71

). Ethical approval for the study was given by the Manchester Local Research Ethics Committees. Women with any history of cervical intraepithelial neoplasia (CIN) or with a previous total hysterectomy were excluded from the study.

### Focus groups

Eight focus groups were conducted (two per ethnic group, each group met only once), during July and September 2000, in English, Gujarati or Urdu as appropriate. The group discussions were tape-recorded, transcribed verbatim and translated into English where necessary. To ensure that all participants had the same baseline knowledge, basic information about cervical cancer and screening, and detailed information about HPV testing was provided at the beginning of the discussion session. Information about HPV concerned high-risk types associated with cervical cancer and covered viral transmission, latency, prevalence and association with CIN, and included information about the potential utility of HPV testing within primary cervical screening. All information was approved by clinical specialists (AS and MD). Core questions explored attitudes towards HPV testing covering: reactions to HPV as an STI linked to cervical cancer; anticipated reactions to testing positive for HPV; partner, family and community attitudes to HPV testing, and religious and cultural influences.

### Analysis

Data were analysed using framework analysis (Ritchie and Spencer, 1994). This is a matrix-based approach to qualitative data analysis. The transcripts were examined by two authors (KM and SF) to identify emergent themes and to develop the framework analytic structure. The FA technique involves identifying recurring and important themes based on a combination of *a priori* issues, emergent issues and recurring attitudes or experiences. Once the first themes are identified they are applied to further transcripts where they are refined so that they encapsulate the diversity of participants' experiences.

## Results

### Upset, stigma and confusion

Women expressed ‘worry’, ‘shock’, ‘surprise’ and even ‘fear’ in response to the information that cervical cancer was linked to a sexually transmitted virus. Responses were set against a background in which cervical cancer itself was perceived as a ‘serious’ and frightening disease. Few women were fully aware of the sexually transmitted nature of cervical cancer. There was confusion between high-risk HPV types (often called the ‘wart virus’ by medical professionals) and other HPV types such as genital warts or plantar warts. Women described concern about transmission of infection from warts on hands to the genital and cervical area during sex, and questioned the impact of these and other HPV types (e.g. genital warts) to their risk of CIN.

The stigma associated with STIs was implicit in women's dramatic reactions to HPV testing and was similar across all of the ethnic groups studied. Reactions such as ‘I would cry for a month’ and ‘panic’ indicate the strong negative emotional response to HPV as an STI. Stigma was explicitly mentioned by some women, who highlighted the negative connotations of the term ‘wart’, particularly its link with genital warts (symptomatic types, visible to the naked eye), which seemed to have a long-standing stigma as an STI, and also with other types of wart. ‘Warts’ in any form were viewed as unpleasant and unwanted, and these perceptions were described as being rooted in early childhood experience that involved others avoiding contact and exclusion from physical education activities carried out barefoot (Box 1

**Box 1:** Reactions to HPV information


*HPV as an STI*
‘Women will get frightened and scared. The word cancer is scary enough and now this new wart virus will cause many of us to be wary’

*Confusion*
‘If somebody has warts, whatever sort…are you at any more risk of this particular virus…they’re all from the same family and if somebody has this little thing on his finger?’

*Stigma*
‘I will be heartbroken and worried for my health and for my family’
‘I think warts in general have a cross on them’

).

### Impact of testing positive for HPV

Women were asked how they would feel if they tested positive for HPV. Participants in all groups described their reactions in terms of anger, distress and anxiety. They also raised questions such as *who* the infection came from, and *when*. Some women also demonstrated more positive views about testing, including feeling relieved that ‘something’ could be found at an ‘early stage’, which was viewed as more amenable to effective treatment. Some respondents balanced the psychological costs of testing, in terms of anxiety, distress and worries about their relationship, against the physical benefits of efficient early detection, and the psychological benefits of reassurance following testing. These more positive views were only expressed by women in the African-Caribbean and white British groups. In the Indian and Pakistani groups, emphasis was given to the psychological costs of screening in terms of anxiety and stress, and relationship difficulties following a positive result (Box 2

**Box 2:** Testing positive for HPV


*Reactions to results*
‘After the initial shock you will wonder how and when it happened and was it before your marriage?’

‘It would reassure me I suppose, if I hadn’t had it done, I wouldn’t know if I’d got it and it could be another 3–4 years before I had my smear done. So I’m closer to having treatment’

*Trust*
‘I will mistrust my husband’
‘It makes you suspicious of him’

*Blame*
‘You will feel like killing him’
‘If I tested positive, I would worry who I had slept with’
‘My husband will deny giving it to me and will accuse me of sleeping with someone else’

*Test of fidelity*
‘If my partner was cheating on me he would discourage me [from being tested]’
‘This will be a good way of testing how much they [husbands] care about us’

*Protection*
‘We will need to protect ourselves’
‘My husband will get angry if I ask him to wear condoms. He will think I am doubting him’

).

Testing positive for HPV raised challenging issues concerning participants’ relationships. Issues of trust, blame, sex and fidelity, and protection emerged as key themes in women's explanations. These themes featured in the accounts of women from all ethnic and socioeconomic groups.

(i) *Trust*. Trust emerged strongly with deep concerns expressed over trust being ‘broken’ between women and their sexual partners/spouses.

(ii) *Blame*. Who was to blame for the infection dominated responses to testing HPV positive. Women described anticipating feelings of ‘anger’, ‘hurt’ and ‘suspicion’ towards their current sexual partner and questioned the history of previous sexual partners. Women, particularly those in the Indian and Pakistani groups, also expressed serious concern and, in some cases, fear that their partner might attribute the blame for HPV infection to them, irrespective of their own previous sexual behaviour.

(iii) *A test of fidelity*. It was suggested by women in the Indian and Pakistani groups that the test might be used as a test of faithfulness, ‘we will know if our husbands are being faithful or not’, and an indicator of how much their partners ‘care’.

(iv) *Protection*. Among women in all the ethnic groups studied, the prospect of testing HPV positive raised questions about protection and safe sex behaviour. Women were told in the study information that protection offered by condoms was uncertain, yet they still identified condoms as the means to protect themselves from HPV. The prospect of using condoms raised worries about the complexities involved in suggesting condom use to partners, which could be perceived as indicating ‘mistrust’ and ‘suspicion’, particularly among women with long-term partners/spouses. It was also suggested that becoming HPV positive, after making a deliberate and emotionally costly effort to use condoms, would add to women's feelings of frustration and helplessness. Abstaining from sex completely was suggested by a few women as a possible strategy for preventing HPV: ‘why bother to have sex when you know it may cause you a problem?’

### Communicating unwanted messages

Attending for HPV testing and testing positive for HPV were identified as potentially communicating unwanted messages to one's partner, family and community.

#### Mistrust and infidelity

Women from all the groups, but notably Indian and Pakistani women, highlighted the messages of distrust or infidelity that may be conveyed to one's partner by simply attending screening, particularly for women in long-term monogamous relationships.

#### Promiscuity

Attending screening or testing positive for HPV was also suggested as implying promiscuity and sexual activity to others such as family (particularly parents) and friends, for women in all the groups. Women from the Indian and Pakistani groups suggested that there may be denial that HPV testing is necessary, with their communities viewed as not at risk of sexually transmitted disease. However, such views were not considered to be held universally and were attributed to older family or community members and less relevant for ‘educated’ men and women.

#### Nontraditional beliefs and practices

For the Indian and Pakistani women, testing was also perceived to reflect nontraditional cultural or religious practices concerning sex and monogamy to their family and community. Muslim beliefs were described by some of the Pakistani women as potentially prohibitive of screening, drawing on examples of the limits imposed on abortion, women visiting male doctors and concerns that cervical screening may affect unmarried women's virginity, with Shari’a law^1^ identified as particularly restrictive. However, others felt that Islamic beliefs would support HPV screening as good for women's health, and hence family health. Among the white British women, taboos surrounding sex within Catholic families were also raised as potentially restrictive to women participating in HPV testing (Box 3

**Box 3:** Communicating unwanted messages


*Mistrust*
‘My partner would see no point [in testing] because he has been my only partner’ (ndian)

*Promiscuity*
*Women reporting a friend's experience of an abnormal smear indicating HPV infection*
‘3 weeks of articles in [popular women's magazine.. very misleading, sayig that the number of partners you have, you are more or less going to develop cancer.. that was a friend of mine who is now 47 and her mum.. had given her that article.. she said you know I had less than 5 partners, I've always used condoms and my mother is thinking I'm a red woman’ (White, British)

*Nontraditional beliefs and practices*
‘My family would see no point to it as you only have one partner’ (Pakistani)
‘Being single, my family will be suspicious of me [if I go for HPV testing]’ (Pakistani)

).

### Importance of information about HPV

There was a universal expression of the need for more information about HPV: ‘many women need to know this information’. The belief that women ‘should’ have information was forcefully expressed with a desire for a better understanding of disease risk and ‘transmission’, to enable women to make ‘choices and changes in their lifestyles’. The recognition that HPV testing had implications beyond the individual led to the request for information to be provided not only for women, but for men and also the wider community: ‘we need to educate the community’. It was recognised, in all groups, that clear and accurate information was critical to everyone's response to HPV and the potential impact of HPV testing. The current lack of accessible information about HPV and its potential to lead to problems was also raised: ‘ignorance causes many problems’.

## Discussion

These findings highlight some of the complex issues that may be raised by the introduction of HPV testing in cervical screening. A positive test result was anticipated to lead to considerable anxiety and distress, and HPV was stigmatised as an STI and associated with genital warts, which held their own long-standing stigma. There was confusion between high-risk HPV, genital warts and other low-risk wart types, and concern about cervical cancer risk and transmission. This has practical implications for giving information to women and suggests that the use of the term ‘wart virus’ to describe high-risk HPV, a common practice among health professionals, may encourage confusion and exacerbate stigma.

The prospect of testing positive for HPV raised important issues for women's relationships in terms of trust, blame, fidelity and protection through safer sex, and testing had the potential to communicate unwanted messages to one's partner, family and the wider community about trust and sexual behaviour. Concerns were expressed by women from all of the ethnic groups, but appeared to be particularly pertinent for some of the Indian and Pakistani women, for whom sex outside marriage can be strongly proscribed. In such circumstances, HPV infection (or perceived risk of infection) would have more significant consequences since it could not be openly attributed to sexual partners encountered prior to marriage. These findings have serious implications particularly among some South Asian women, whose chosen sexual lifestyle may directly impact on their family and wider community (Bradbury and Williams, 1999; Elam *et al*, 1999). However, among all participants, irrespective of ethnic and socioeconomic group, relationship status and history appeared to play a pivotal role in the anticipated impact of testing positive for HPV.

Of both practical and theoretical importance, women in all groups demonstrated confusion about condom use and protection from HPV. Despite explanations that evidence for the efficacy of condoms in preventing HPV transmission is uncertain (CDC, 1999; Carr and Gyorfi, 2000), they were still viewed by some women as the means to *ensure* prevention of infection. This suggests that condoms may be perceived as a method of preventing *any* STI, perhaps following HIV health promotion (Gardner *et al*, 1999). Health professionals may need to find new ways to communicate information about the ambiguity surrounding condom use within the context of HPV prevention.

Despite the anticipated negative consequences of HPV testing, women from the white British and African-Caribbean groups in the sample also expressed positive views about testing, and welcomed the added protection that HPV testing could offer in terms of cervical cancer prevention. Women from all groups identified existing information about HPV as inadequate, and highlighted that the reaction to HPV testing would be determined by the effectiveness of health information about the virus, its transmission, latency, prevention and association with CIN. Such information, while being difficult to communicate, may be critical to manage the potentially negative impact of HPV.

Our findings have implications for the management of HPV testing within cervical screening. However it is important to note that none of the women involved in the study were offered HPV tests or had previously participated in HPV testing. Women responded to new information about the link between HPV and cervical cancer. However, the study is advantaged by its ethnically heterogeneous sample and the inclusion of older married women, who have been poorly represented in previous research, but for whom the impact of HPV may be greater, since they are more likely to be in long-term monogamous relationships. Previous work on the psychosocial impact of HPV testing has been quantitative and sampled young, white, affluent populations, often American college students (Ramirez *et al*, 1997; Reed *et al*, 1999), and has failed to distinguish psychological outcomes between those with visible, symptomatic genital warts (low-risk types) and those with high-risk HPV (Maw *et al*, 1998). The current study overcame both these limitations.

Study participants were purposively selected to represent heterogeneity of attitudes and experiences to HPV and cervical screening relating to their ethnic, socioeconomic and sexual relationship background, and this paper sought to illustrate the key themes that emerged across the sample. As is usual in qualitative research, participants were not selected to be statistically representative of their ethnic and socioeconomic group; rather, they were selected to represent a range of women eligible for cervical screening so that a diversity of experiences and attitudes could be covered. Almost all women had participated in screening previously (93%, *n=*66), which compares to 90% within the national population (Cervical Screening Programme annual review 2002, England). Although some categories of women were numerically under-represented in the study sample, the full range of women, and their views, were represented.

Although the study is qualitative and our sample was purposively selected, its findings may be generalised beyond the study population (Morse, 1999). The findings suggest that the psychosocial consequences of HPV testing may be negative for some women, their partners and families, and this impact may relate to ethnic, cultural and religious background, and sexual relationship status and history. Research is now needed to quantify this impact among representative samples within the UK population who are tested for HPV infection. The possible consequences we observed hold implications not only for the introduction of HPV testing, but also for current information provision for women undergoing cervical screening in Britain. Whether HPV testing is introduced or not, awareness of the association between cervical cancer and HPV is probably inevitable. Our research identifies a critical need for accurate information accessible to all ethnic groups prior to participation in cervical screening. Women clearly felt that they and, importantly, those around them should have access to more information about their own risk, managing this risk and interpreting positive results. The current management of HPV within cervical screening raises uncomfortable questions about informed participation in cervical screening and the ethics of current practices, which seek to maintain high levels of screening uptake. Our findings add support to the growing movement towards more informed choice at screening, whether it be cytology or HPV, as the negative psychosocial costs of participation could, for some women, be substantial.

## Footnotes

1 Shari'a (Islamic law) is derived directly from the Qu’ran and sets out a legal system, which governs individual and social life. The interpretation of Shari’a principles and practices vary considerably both among and within Muslim societies and communities.
